# Biochemotherapy of metastatic malignant melanoma. Predictive value of tumour-infiltrating lymphocytes

**DOI:** 10.1054/bjoc.2001.2169

**Published:** 2001-12

**Authors:** A Håkansson, B Gustafsson, L Krysander, B Hjelmqvist, B Rettrup, L Håkansson

**Affiliations:** Department of Oncology, Department of Pathology and Cytology, Department of Hand and Plastic Surgery, University Hospital, Linköping, SE-581 85; Department of Surgery, County Hospital, Kalmar, SE-391 85, Sweden

**Keywords:** malignant melanoma, biochemotherapy, prediction of response, tumour-infiltrating lymphocytes (TILs)

## Abstract

The therapeutic efficacy of biochemotherapy in metastatic malignant melanoma still carries a low remission rate, but with some durable responses. It would therefore be of considerable importance if patients with a high probability of responding could be identified using predictive tests. The response to interferon-alpha (IFN-α) correlates with the occurrence of CD4^+^ lymphocytes identified by fine-needle aspirates from melanoma metastases (Håkansson et al, 1996). The present investigation studies a possible correlation between tumour-infiltrating CD4^+^ lymphocytes in malignant melanoma metastases and the therapeutic effect of biochemotherapy. A total of 25 patients with systemic and 16 with regional metastatic melanoma were analysed before initiation of biochemotherapy (cis-platinum 30 mg/m^2^ d.1–3, DTIC 250 mg/m^2^ d.1–3 i.v. and IFN-α2b 10 million IU s.c. 3 days a week, q. 28d.). A monoclonal antibody, anti-CD4, was used to identify tumour-infiltrating lymphocytes in fine-needle aspirates before start of treatment. The presence of these lymphocytes was correlated to response, time to progression and overall survival. A statistically significant correlation (*P* = 0.01) was found between the occurrence of CD4^+^ lymphocytes and tumour regression during biochemotherapy in patients with systemic disease. Out of 14 patients with moderate to high numbers of infiltrating CD4^+^ lymphocytes, 12 achieved tumour regression. In contrast, among patients with low numbers of these cells in metastatic lesions, 8 out of 11 had progressive disease. We also found a significantly longer time to progression (*P* < 0.003) and overall survival (*P* < 0.01) among patients with moderate to high numbers of these cells compared to patients with low numbers of these cells before initiation of biochemotherapy. Furthermore, in patients with regional disease, we found a significantly longer time to progression (*P* = 0.01) and a trend toward a longer overall survival time (*P* = 0.09). Based on these results and as previously shown with IFN-α therapy alone, there seems to be a need for CD4^+^ lymphocytes infiltrating the tumours before the start of biochemotherapy to make the treatment successful. Determination of these cells in fine-needle aspirates seems to be a method to predict responders to biochemotherapy, thus increasing the cost–benefit of this treatment strategy considerably, both in terms of patient adverse reactions and health care costs. © 2001 Cancer Research Campaign http://www.bjcancer.com


British Journal of Cancer (2001) **85**(12), 1871–1877
© 2001 Cancer Research Campaign
doi: 10.1054/ bjoc.2001.2169, available online at http://www.idealibrary.com on IDEAL
http://www.bjcancer.com

### Biochemotherapy of metastatic malignant melanoma. Predictive value of tumour-infiltrating lymphocytes

**A Håkansson[1], B Gustafsson[2], L Krysander[3], B Hjelmqvist[4], B Rettrup[4] and L Håkansson[1]**

[1]Department of Oncology; [2]Department of Pathology and Cytology; [3]Department of Hand and Plastic Surgery, University Hospital, SE-581 85 Linköping; [4]Department of Surgery, County Hospital, SE-391 85 Kalmar, Sweden

**Summary** The therapeutic efficacy of biochemotherapy in metastatic malignant melanoma still carries a low remission rate, but with some durable responses. It would therefore be of considerable importance if patients with a high probability of responding could be identified using predictive tests. The response to interferon-alpha (IFN-α) correlates with the occurrence of $CD4^+$ lymphocytes identified by fine-needle aspirates from melanoma metastases (Håkansson et al, 1996). The present investigation studies a possible correlation between tumour-infiltrating $CD4^+$ lymphocytes in malignant melanoma metastases and the therapeutic effect of biochemotherapy. A total of 25 patients with systemic and 16 with regional metastatic melanoma were analysed before initiation of biochemotherapy (cis-platinum 30 mg/m$^2$ d.1–3, DTIC 250 mg/m$^2$ d.1–3 i.v. and IFN-α2b 10 million IU s.c. 3 days a week, q. 28d.). A monoclonal antibody, anti-CD4, was used to identify tumour-infiltrating lymphocytes in fine-needle aspirates before start of treatment. The presence of these lymphocytes was correlated to response, time to progression and overall survival. A statistically significant correlation ($P$ = 0.01) was found between the occurrence of $CD4^+$ lymphocytes and tumour regression during biochemotherapy in patients with systemic disease. Out of 14 patients with moderate to high numbers of infiltrating $CD4^+$ lymphocytes, 12 achieved tumour regression. In contrast, among patients with low numbers of these cells in metastatic lesions, 8 out of 11 had progressive disease. We also found a significantly longer time to progression ($P$ < 0.003) and overall survival ($P$ < 0.01) among patients with moderate to high numbers of these cells compared to patients with low numbers of these cells before initiation of biochemotherapy. Furthermore, in patients with regional disease, we found a significantly longer time to progression ($P$ = 0.01) and a trend toward a longer overall survival time ($P$ = 0.09). Based on these results and as previously shown with IFN-α therapy alone, there seems to be a need for $CD4^+$ lymphocytes infiltrating the tumours before the start of biochemotherapy to make the treatment successful. Determination of these cells in fine-needle aspirates seems to be a method to predict responders to biochemotherapy, thus increasing the cost–benefit of this treatment strategy considerably, both in terms of patient adverse reactions and health care costs. © 2001 Cancer Research Campaign http://www.bjcancer.com

**Keywords**: malignant melanoma; biochemotherapy; prediction of response; tumour-infiltrating lymphocytes (TILs)

Despite multiple approaches to treating metastatic malignant melanoma, stage IV disease is still associated with a poor prognosis. The most commonly used single agent, dacarbazine (DTIC), produces a response in the range of 15–20% (Houghton et al, 1992). Combination chemotherapy has yielded higher response rates but predominantly partial remissions and of short duration (Legha et al, 1989; McClay et al, 1992). Biological agents such as interleukin-2 (IL-2) and interferon-alpha (IFN-α) have been studied extensively. Response rates have been in the same range as with DTIC but in contrast to responses achieved with chemotherapy, a subset of responses with biological agents is more durable (Legha, 1997). In recent years several groups have reported their experiences with combination treatment, biochemotherapy (Legha, 1997; Khayat et al, 1993; Keilholz et al, 1998; Proebstle et al, 1998; Richards et al, 1999; Rosenberg et al, 1999). Unfortunately, many patients do not benefit, whereas others have responses, some of which are durable and thus very valuable. It would therefore be of considerable importance if patients with a high probability of responding could be identified before the start of treatment using predictive tests.

As biological agents modulate the activity of various cells in the immune system, it is reasonable to assume that the therapeutic effect might depend on the immune status of the patient when therapy is initiated. Several groups have reported on the importance of tumour-infiltrating lymphocytes in primary (Clark et al, 1989; Clemente et al, 1996) and metastatic malignant melanoma (Mihm et al, 1996; Zehntner et al, 1999). Despite some conflicting results (Balch et al, 1978), lymphocyte infiltration in primary malignant melanoma seems to be of prognostic significance (Clark et al, 1989; Clemente et al, 1996; Hansen and McCarten 1974; Larsen et al, 1978). In regressing melanomas, a predominance of $CD4^+$ cells has been found (Halliday et al, 1995; Tefany et al, 1991). In metastatic disease, a high CD4/CD8 ratio has indicated a more favourable prognosis (Hernberg et al, 1997). Our group has previously shown the importance of $CD4^+$ lymphocytes in melanoma metastases for response to IFN-α treatment (Håkansson et al, 1996). With granulocyte macrophage colony stimulating factor (GM-CSF) treatment, responding patients showed marked increases and high absolute numbers of T-cell infiltrates into the tumour, particularly of the CD4 T-cell subset (Si et al, 1996). This also concurs well with preliminary data from Zehnenter et al (1999) suggesting that T-cell infiltration is associated with clinical response to melanoma immunotherapy.

Received 15 April 2001
Revised 18 September 2001
Accepted 13 October 2001

Correspondence to: A Håkansson

The aim of the present investigation was therefore to study whether the presence of tumour-infiltrating CD4$^+$ lymphocytes identified by fine-needle aspirates of melanoma metastases before treatment is started also correlates with the response to biochemotherapy.

### MATERIALS AND METHODS

### Study population

This report describes 41 patients with metastatic malignant melanoma, 26 males and 15 females. The median age was 61 years (range 36–77) and Karnofsky performance status was 70 or more. Recurrences were cytologically verified by fine-needle aspirates before start of treatment. Two groups of patients were studied: those with regional disease and those with systemic disease. A total of 16 patients had regional metastases and 25 patients had systemic disease with the following metastatic sites: 18 lymph nodes, 8 subcutaneous, 2 cutaneous, 11 lung, 5 liver, 4 spleen, 1 bone, 1 adrenal gland and 2 soft tissue metastases. The number of metastatic sites in patients with systemic disease was 1 in 9 patients, 2 in 9 patients, 3 in 3 patients and 4 in 4 patients. In the group with regional metastases in two patients, it was the first recurrence, in 11 patients, the second recurrence, in 2 patients, the third and in 1 patient, the fourth regional recurrence. Patients with symptoms of brain metastases were not included in this study.

### Pre-treatment investigations and treatment schedule

Pre-treatment investigations included electrocardiogram (ECG), abdomina computerized tomography (CT), chest X-ray and blood samples for measurements of creatinine, bilirubin, alkaline phosphatase, alanine aminotransferase, lactate dehydrogenase, alpha amylase, haemoglobin, white blood cells, and thrombocytes. The treatment schedule was cisplatinum 30 mg/m$^2$ d.1–3 i.v., DTIC 250 mg/m$^2$ d.1–3 i.v. and IFN-α2b 10 million IU s.c. 3 days a week. The duration of one cycle was 28 days. One patient also received IL-2 with cycle 1, and two patients were on tamoxifen treatment.

### Fine-needle aspiration of metastases and immunological staining

Aspirates were taken from the tumour with a 0.6 mm hypodermic needle. Only one site and tumour had a fine-needle aspiration in each patient. The aspirate was smeared on a glass slide and was allowed to dry in air. At least two smears were then stained for conventional cytomorphology according to the May–Grunewald–Giemsa method. In cases without obvious melanin pigment in tumour cells, the diagnosis of melanoma was confirmed with immunostaining for vimentin and protein S-100. Morphological signs of degeneration or necrosis were registered.

The slides were air dried and then fixed for 5 min in acetone. After drying, the slides were washed in phosphate-buffered saline (PBS), pH 7.6, and incubated with the monoclonal antibody against CD4 for 30 min (Becton-Dickinson, Stockholm, Sweden). Mouse IgG (Sigma, Stockholm, Sweden) was used as a negative control. After washing in PBS, the sections were incubated with rabbit anti-mouse immunoglobulin (Dakopatts, Z 259) for 30 min, washed in PBS and incubated with the PAP mouse monoclonal antibody (Dakopatts, P 850) for 30 min. After washing in PBS, the slides were incubated in 50 ml PBS containing 40 mg Diaminobenzidin (DAB, Sigma, Stockholm, Sweden) and 0.6 ml 3% $H_2O_2$ for 6 min and washed. The slides were counterstained in Mayers haematoxylin for 15 min, washed and mounted in Glycergel (Dakopatts, Sweden). All incubations were performed in a moist chamber.

### Evaluation of mononuclear cells

The percentage of CD4$^+$ lymphocytes was counted at a magnification of 400 × using a square inserted into the ocular. In total, 500 cells were counted in randomly selected areas and the mean of these counts was used for statistical analysis. When the percentage of lymphocytes was more than 2%, it was scored as medium/high; a percentage less than 2% was scored as low. CD4$^+$ cells scored as lymphocytes had small or medium-sized nuclei and sparse cytoplasm with distinct cell borders. Macrophages displayed large nuclei and abundant, generally faintly stained cytoplasm. The proportion of lymphocytic vs non-lymphocytic CD4$^+$ cells was registered.

### Preparation of tumour biopsies and immunological staining of tissue sections

Biopsies from resected tumours were immediately snap-frozen and stored at –70˚C until further processing. Tissue sections were later prepared as described previously (Håkansson et al, 1996). The degree of histopathological tumour regression was evaluated on negative controls, not incubated with specific primary antibodies.

### Criteria of response in patients with systemic disease (according to WHO)

A complete response (CR) was defined as a disappearance of all known disease. *A* partial response (PR) was defined as a decrease by at least 50% in the sum of the products of the largest perpendicular diameters of measurable lesions determined by two observations not closer than 4 weeks apart. It is not necessary for all lesions to have regressed for the patient to be classified as having a partial response, but no lesion should have progressed and no new lesions should have appeared. Minor regressions did not fulfil the criteria for partial regression, either because the reduction in the tumour size was 25–50% or the duration of the response was too short. A mixed response was defined as a measurable shrinkage of some lesion and simultaneous progressive disease in some other metastasis, or the appearance of new lesions. Stable disease (SD) was defined as a 25% decrease in total tumour size could not be established nor has a 25% increase in the size of one or more measurable lesions been demonstrated. In addition, patients with stable disease will have no appearance of new lesions. Progressive disease (PD) is defined as a 25% or more increase in the size of at least one measurable lesion or the appearance of a new lesion. As the objective of this study was to analyze a correlation between the occurrence of tumour-infiltrating CD4$^+$ lymphocytes and the anti-tumour effect of biochemotherapy, significant tumour regression (more than 25%) in patients with minor regressions and mixed responses, not fulfilling the formal criteria for partial remission, were used in the following analysis.

### Evaluation of tumour regression in patients with regional metastases and criteria of histopathological tumour regression

Among patients with regional resectable disease, clinical tumour response may be difficult to detect based on tumour size only.

 *© 2001 Cancer Research Campaign*

Instead, the occurrence of tumour regression was evaluated by histopathological examination of tumour biopsies. Based on the description of regressive changes in primary malignant melanoma in other studies and as previously described by our group (Håkansson et al, 1996; McGovern, 1975; Kang et al, 1993; Sondergard and Hou-Jensen, 1985; Ronan et al, 1987) the following criteria of tumour regression were used in this study:

1. Low and variable density of tumour cells, particularly variation in density within the same tumour nodule
2. Disorganization of the architecture of the tumour with nests of remaining tumour cells surrounded by stromal tissue
3. Fibrosis.

However, the inflammatory infiltrate was not used as a criterion of histopathological tumour regression in this study. The signs of regression vary from no signs to almost complete destruction with only few tumour cells present. The degree of tumour regression was considered minor when regressive changes were estimated to be less than 25% (minor regression) and marked when such changes were estimated to be more than 25% (marked regression) of the section area. Tumour regression areas of more than 25% are generally not found in tumour biopsies from untreated patients (Håkansson et al, 1998).

### Statistical analyses

The differences in distribution of inflammatory cells between patients with tumour response and progressive disease and between patients with marked and minor or no histopathological tumour regression were analyzed using the $\chi^2$ test.

Survival curves were plotted using the Kaplan–Meier method, and time to progression and survival comparisons between subgroups were performed using the log rank test.

The study was approved by the ethical committee at the University Hospital of Linköping, Sweden.

### RESULTS

### Correlation between treatment efficacy and occurrence of tumour-infiltrating mononuclear cells in patients with systemic disease

Reduction in tumour size after biochemotherapy was registered in 15 out of 25 patients with systemic disease. Of these 25, 2 had a complete remission, 8 had partial remission, 4 had a reduction of measurable tumours of 25–50%, 1 had a mixed response, 1 had stable disease and 9 patients had progressive disease. The overall objective response rate was 40% and 60% of patients achieved at least a minor or mixed response. The 2 patients who achieved complete remission later had their treatment changed from biochemotherapy to IL-2 because of adverse side effects. These changes in both patients were made after they already had achieved complete remission on the biochemotherapy regimen. One had progressive disease and died after 3 months, the other is still disease-free after 42 months. Two patients with partial remission and 1 patient with a minor response after biochemotherapy achieved complete remission after surgery; 2 had recurrent disease after 7 and 13 months respectively, and one is still disease-free.

In order to register any capacity to respond to biochemotherapy, mixed responses and minor regressions not fulfilling the formal criteria for partial remission were included in the analysis. The occurrence of $CD4^+$ lymphocytes in fine-needle aspirates (Figure 1) in relation to the therapeutic effect of biochemotherapy

**Table 1** Pre-treatment tumour-infiltrating CD4+ lymphocytes in fine-needle aspirates from patients with systemic disease according to clinical effect of biochemotherapy

| Proportion of infiltrating cells | No. of patients with tumour-infiltrating CD4+ lymphocytes according to tumour response | | |
|---|---|---|---|
| | Regression | Stable disease | Progressive disease |
| Low | 3 | 0 | 8 |
| Moderate/high | 12 | 1 | 1 |

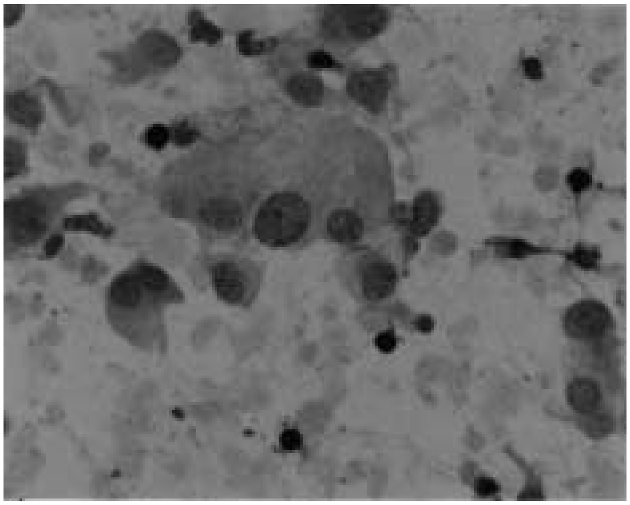

**Figure 1** Occurrence of tumour-infiltrating $CD4^+$ lymphocytes in a fine-needle aspirate from a melanoma metastases before initiation of biochemotherapy

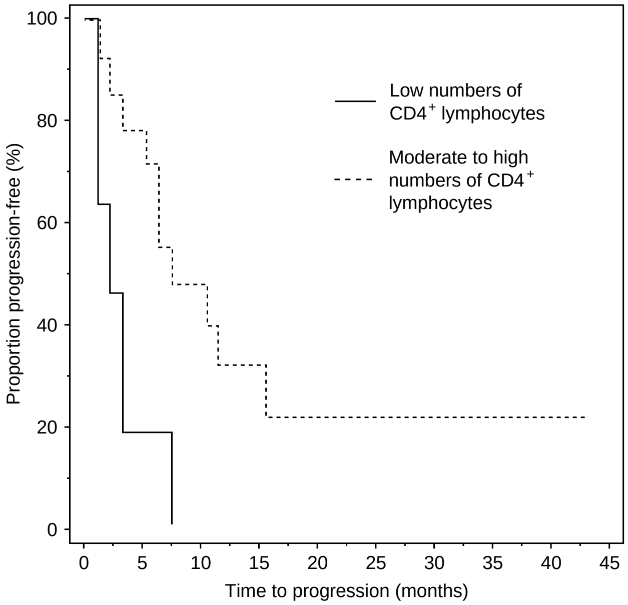

**Figure 2** Time to progression in patients with systemic disease according to the number of tumour-infiltrating $CD4^+$ lymphocytes before initiation of biochemotherapy, $P < 0.003$

*© 2001 Cancer Research Campaign*

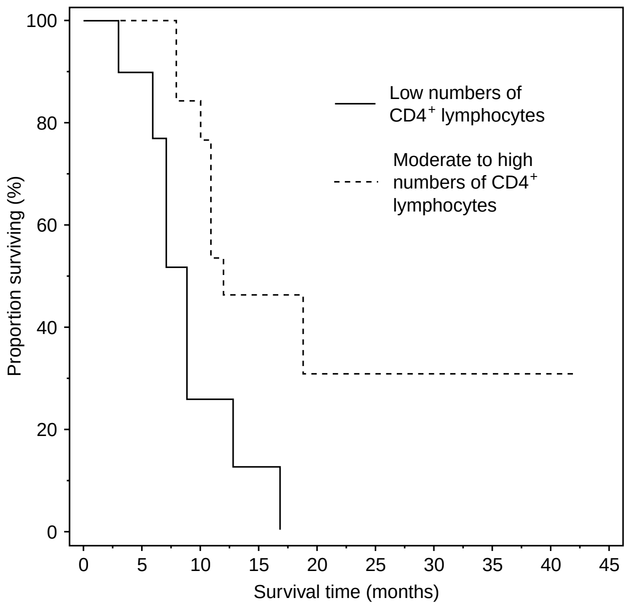

**Figure 3** Overall survival in patients with systemic disease according to the number of tumour-infiltrating CD4+ lymphocytes before initiation of biochemotherapy, $P < 0.01$

is shown in Table 1. Obviously there is a close correlation between the anti-tumour effect and presence of CD4+ lymphocytes. Out of 25 patients, 14 had moderate/high numbers of tumour-infiltrating CD4+ lymphocytes and 12 out of 14 achieved tumour regression. In contrast, 8 out of 11 patients with low numbers of these cells had progressive disease ($P = 0.01$).

We also found a significantly longer time to progression ($P < 0.003$) and overall survival ($P < 0.01$) among patients with moderate/high numbers of CD4+ lymphocytes compared to patients with low numbers of these cells before initiation of biochemotherapy (Figures 2 and 3).

### Correlation between treatment efficacy and occurrence of tumour-infiltrating mononuclear cells in patients with regional metastases

Among patients with regional metastases, it was difficult to adequately determine treatment efficacy based on tumour size only, as the majority of these patients had their metastases resected after the first treatment cycle. Therefore, histopathological criteria used for tumour regression were applied in the evaluation. A total of 10 patients had marked histopathological regression (Figure 4A) of the resected metastases and 4 patients had only minor or no regressive changes (Figure 4B). After treatment, 2 patients did not undergo surgery 1 because of extensive progression of the disease and 1 because of difficulties in performing more extensive surgery after previous resections.

Although the number of patients with regional metastases is small, a similar tendency to that of patients with systemic disease was found. Of 8 patients with moderate/high numbers of tumour-infiltrating CD4+ lymphocytes, 7 showed marked histopathological regression, in contrast to 3 out of 6 patients with low numbers of these cells in the metastases.

Moreover in patients with regional disease, we found a significantly longer time to progression ($P = 0.01$) and a trend toward a longer overall survival ($P = 0.09$) among patients with moderate/high numbers of CD4+ lymphocytes compared with patients with low numbers of these cells before initiation of biochemotherapy (Figures 5 and 6).

### DISCUSSION

In recent years, biochemotherapy has been used by many groups treating metastatic malignant melanoma (Legha, 1997; Khayat et al, 1993; Keilholz et al, 1998; Proebstle et al, 1998, Richards et al, 1999; Rosenberg et al, 1999). A subset of durable, and thus very valuable, remissions has been shown compared with chemotherapy alone. As treatment, however, is associated with significant toxicity and many patients still do not benefit, there is a great need for predictive tests identifying patients with a high probability of response.

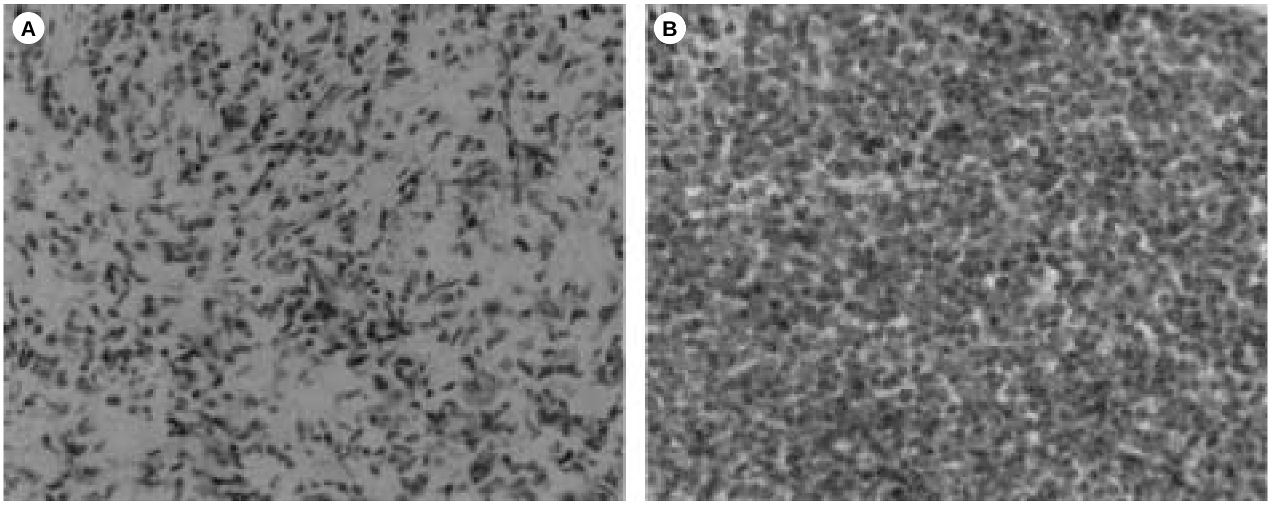

**Figure 4** (**A**) Marked tumour regression in a tumour biopsy after biochemotherapy. (**B**) Tumour biopsy without regressive changes after biochemotherapy

 © 2001 Cancer Research Campaign

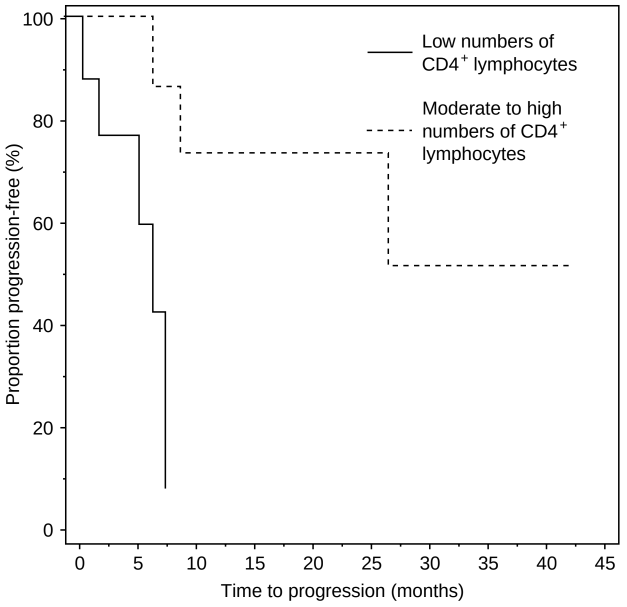

**Figure 5** Time to progression in patients with regional disease according to the number of tumour-infiltrating CD4+ lymphocytes before initiation of biochemotherapy, $P$ = 0.01

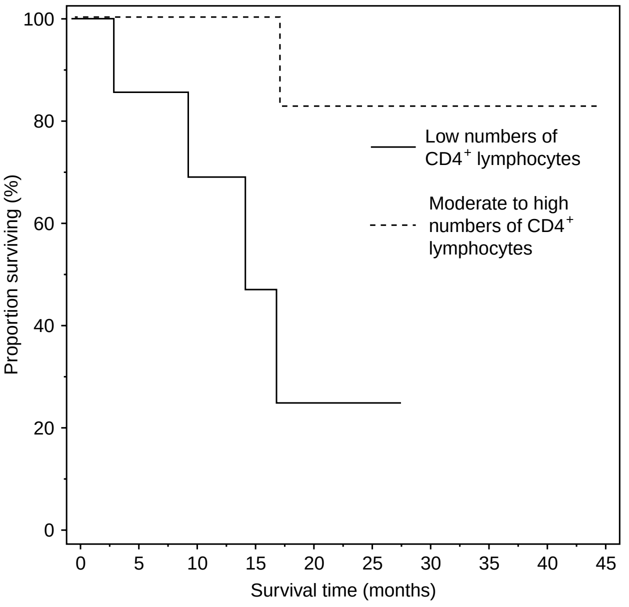

**Figure 6** Overall survival in patients with regional disease according to the number of tumour-infiltrating CD4+ lymphocytes before initiation of biochemotherapy, $P$ = 0.09

In the adjuvant setting also, immunotherapy has shown important results on relapse-free (Pehamberger et al, 1998; Grob et al, 1998; Kirkwood et al, 2000) and overall survival (Kirkwood et al, 1996). Today, several different studies are undertaken, where immunotherapy is given to high-risk patients. Also in this setting, however, many patients do not benefit. Because of this, there is a need for a predictive test that selects patients who are suitable for this kind of therapy.

Biological agents modulate various functions in the immune system. It is thus reasonable to assume that the therapeutic effect might depend on the presence of certain subsets of mononuclear cells infiltrating the tumours before treatment is initiated. Therefore the present study analysed the presence of tumour-infiltrating lymphocytes in melanoma metastases before the start of biochemotherapy.

As has previously been shown with IFN-α therapy (Håkansson et al, 1996), the present study demonstrated a close correlation between the occurrence of CD4+ lymphocytes and the therapeutic benefit of biochemotherapy with IFN-α, DTIC and cisplatinum. In patients with systemic disease, 12 out of 14 of those with moderate/high infiltration of CD4+ lymphocytes in the tumours achieved tumour regression. In contrast, among patients with low infiltration of these cells, 8 out of 11 had progressive disease. The presence of a moderate/high infiltration of CD4+ lymphocytes in the metastases was also found to correlate with a significantly longer time to progression and a significantly longer overall survival compared to patients with a low infiltration. These results are of great importance as many responses in melanoma treatment are, unfortunately, of short duration and do not show any impact on survival. Thus, the present method not only predicts responders but also identifies patients with a high probability of durable remission.

In order to evaluate properly a possible correlation between response to treatment and a parameter of potential predictive value, it is necessary to include all patients with measurable tumour regression in the analysis, i.e. at least those with minor regression and mixed responses. The reason why short-lived, minor regressions do not continue and develop into partial or complete remissions is not clear, but it is reasonable that the outcome of several months of immunotherapy is the end result of a multi-step process, e.g. initial immune-mediated lysis of tumour cells, selection of non-immunogenic tumour cell clones, and down-regulation of the immune response to the tumour (Håkansson et al, 1999). Thus, in order not to misjudge the possibility of the immune system of these patients responding to immunotherapy, significant minor and mixed responses have to be included in the analyses. If only patients with partial or complete remissions are regarded as responders, other patients with measurable regression will erroneously be allocated to the group of non-responders, which will obscure the results. This misinterpretation of data may be one reason why tests of predictive value for response to immunotherapy have been difficult to find.

Finding immunological tests of predictive value for biochemotherapy might, of course, be even more difficult as some responses seen with combination treatment might depend on the effect of the cytotoxic drugs only. However, a predictive value of the presence of CD4+ tumour-infiltrating lymphocytes was actually found in the present study. The reason for this can be either that patients with an immune status allowing CD4+ lymphocytes to infiltrate into the tumours respond to the type of chemotherapy used in the present study or that there is a synergistic effect between IFN-α and these cytotoxic drugs. It has been demonstrated that cisplatinum can enhance the immune reactivity to the tumour by increasing the expression of Fas (Mizutani et al, 1998) on tumour cells and also inhibiting the production of the IL-1 receptor antagonist (Arenberg et al, 1995).

The need for CD4+ lymphocytes to infiltrate the tumours in order to make the treatment successful agrees well with reports from other groups on the importance of tumour-infiltrating lymphocytes in primary and metastatic melanoma (Clark et al,

*© 2001 Cancer Research Campaign*

1989; Clemente et al, 1996; Mihm et al, 1996; Zehntner et al, 1999; Hernberg et al, 1997; Si et al, 1996).

CD4+ lymphocytes generally have been considered to be immunoregulatory but have, in recent years, also been found to have a direct cytotoxic activity (Thomas and Hersey, 1998). The exact mechanism, however, still remains uncertain. Our group has previously shown that IFN-α treatment facilitates the migration of CD4+ cells from the stroma close to tumour cells and that the extent of the tumour areas with regressive changes was significantly enhanced after IFN-α treatment (Håkansson et al, 1998). These results also support the view that the CD4+ cells interact directly with the tumour cells.

Based on the results with biochemotherapy shown in this study, and as previously shown with IFN-α therapy alone, there is a need for CD4+ lymphocytes to infiltrate the tumours in order to make the treatment successful. Thus, determining tumour-infiltrating CD4+ lymphocytes obtained by fine-needle aspiration seems to be a method of predicting responders. This will increase the cost–benefit of this treatment strategy considerably, both in terms of patient adverse reactions and health care costs.

### ACKNOWLEDGEMENTS

The authors thank Professor John Carstensen, Department of Health and Society, Tema Research Institute for his assistance in the statistical analysis and Karin Hellander and Catharina Tranaeus Röckert for their excellent technical help in performing the immunocyto- and immunohistochemistry stainings. This work was supported by the County Council of Östergötland, by the Health Research Council in the South East of Sweden and by Schering-Plough AB, Stockholm, Sweden.

### REFERENCES

Arenberg DA, Kunkel SL, Burdick MD, Standiford TJ and Strieter RM (1995) Regulation of monocyte derived interleukin 1 receptor antagonist by cisplatinum. *Cytokine* **7**: 89–96

Balch CM, Murad TM, Soong S-J, Ingalls AL, Halpern NB and Maddox WA (1978) A multifactorial analysis of melanoma: Prognostic histopathological features comparing Clark's and Breslow's staging methods. *Ann Surg* **188**: 732–742

Clark WH, Elder DE, Guerry D, Braitman LE, Trock BJ, Schultz D, Synnestvedt M and Halpern AC (1989) Model predicting survival in stage I melanoma based on tumour progression. *J Natl Cancer Inst* **81**: 1893–1904

Clemente CG, Mihm MC, Bufalino R, Zurrida S, Collini P and Cascinelli N (1996) Prognostic value of tumour infiltrating-lymphocytes in the vertical growth phase of primary cutaneous melanoma. *Cancer* **77**: 1303–1310

Grob JJ, Dreno B, Salmoniere P, Delaunay M, Cupissol D, Guillot B, Souteyrand P, Sassolas B, Cesarini J-P, Lionnet S, Lok C, Chastang C and Bonerandi JJ (1998) Randomised trial of interferon α-2a as adjuvant therapy in resected primary melanoma thicker than 1.5 mm without clinically detectable node metastases. *Lancet* **351**: 1905–1910

Halliday GM, Patel A, Hunt MJ, Tefany FJ and Barnetson R St.C (1995) Spontaneous regression of human melanoma/nonmelanoma skin cancer: Association with infiltrating CD4+ T cells. *World J Surg* **19**: 352–358

Hansen MG and McCarten AB (1974) Tumour thickness and lymphocytic infiltration in malignant melanoma of the head and neck. *Am J Surg* **128**: 557–561

Hernberg M, Turunen JP, Muhonen T and Pyrhönen S (1997) Tumour-infiltrating lymphocytes in patients with metastatic melanoma receiving chemoimmunotherapy. *J Immunother* **20**: 488–495

Houghton AN, Legha S and Bajorin DF (1992) Chemotherapy for metastatic melanoma. *Cutaneous Melanoma*. J.B. Lippincott: Philadelphia, pp 498–508

Håkansson A, Gustafsson B, Krysander L and Håkansson L (1996) Tumour-infiltrating lymphocytes in metastatic malignant melanoma and response to interferon-alpha treatment. *Br J Cancer* **74**: 670–676

Håkansson A, Gustafsson B, Krysander L and Håkansson L (1998) Effect of interferon-α on tumour-infiltrating mononuclear cells and regressive changes in metastatic malignant melanoma. *J Interferon Cytokine Res* **18**: 33–39

Håkansson A, Gustafsson B, Krysander L, Hjelmqvist B, Rettrup B and Håkansson L (1999) On down-regulation of the immune response to metastatic malignant melanoma. *Cancer Immunol Immunother* **48**: 253–262

Kang S, Barnhill RL, Mihm MC and Sober AJ (1993) Histologic regression in malignant melanoma: an interobserver concordance study. *J Cutan Pathol* 126–129

Keilholz U, Conradt C, Legha SS, Khayat D, Scheibenbogen C, Thatcher N, Goey SH, Gore M, Dorval T, Hancock B, Punt CJA, Dummer R, Avril MF, Bröcker EB, Benhammouda A, Eggermont AMM and Pritsch M (1998) Results of interleukin-2-based treatment in advanced melanoma: A case record-based analysis of 631 patients. *J Clin Oncol* **16**: 2921–2929

Khayat D, Borel C, Tourani JM, Benhammouda A, Antonie E, Rixe O, Vuillemin E, Bazex PA, Thill L, Franks R, Auclerc G, Soubrane C, Banzet P and Weil M (1993) Sequential chemoimmunotherapy with cisplatin, interleukin-2, and interferon alfa-2a for metastatic malignant melanoma. *J Clin Oncol* **11**: 2173–2180

Kirkwood JM, Hunt Strawderman M, Ernstoff MS, Smith TJ, Borden EC and Blum RH (1996) Interferon alpha-2b adjuvant therapy of high-risk resected cutaneous melanoma: The Eastern Cooperative Oncology Group trial EST 1648. *J Clin Oncol* **14**: 7–17

Kirkwood JM, Ibrahim JG, Sondak VK, Richards J, Flaherty LE, Ernstoff MS, Smith TJ, Rao U, Steele M and Blum RH (2000) High-and low-dose interferon alfa-2b in high-risk melanoma: first analysis of intergroup Trial E1690/S9111/C9190. *J Clin Oncol* **18**(**12**): 2444–2458

Larsen TE and Grude TH (1978) A retrospective histological study of 669 cases of primary cutaneous malignant melanoma in clinical stage I. *Acta Pathol Microbiol Scand* **86**: 523–530

Legha SS, Ring S, Papadopoulos N, Plager C, Chawala S and Benjamin R (1989) A prospective evaluation of a triple drug regimen containing cisplatin, vinblastine, and dacarbazine (CVD) for metastatic melanoma. *Cancer* **64**: 2024–2029

Legha SS (1997) Durable complete responses in metastatic melanoma treated with interleukin-2 in combination with interferon alfa and chemotherapy. *Semin Oncol* **24**(**Suppl 4**) : S39–S43

McClay EF, Mastrangelo MJ, Berd D and Bellet RE (1992) Effective combination chemo/hormonal therapy for malignant melanoma: Experience with three consecutive trials. *Int J Cancer* **50**: 553–556

McGovern VJ (1975) Spontaneous regression of melanoma. *Pathology* **7**: 91–99

Mihm MC, Clemente CG and Cascinelli N (1996) Tumour-infiltrating lymphocytes in lymph node melanoma metastases: A histopathologic prognostic indicator and an expression of local immune response. *Lab Invest* **74**: 43–47

Mizutani Y, Yoshida O and Bonavida B (1998) Sensitization of human bladder cancer cells to Fas-mediated cytotoxicity by cis-diamminedichloroplatinum. *J Urol* **160**: 561–570

Pehamberger H, Soyer P, Steiner A, Kofler R, Binder M, Mischer P, Pachinger W, Auböck J, Fritsch P, Kerl H and Wolff K (1998) Adjuvant interferon α-2a treatment in resected primary stage II cutaneous melanoma. *J Clin Oncol* **16**: 1425–1429

Proebstle TM, Fuchs T, Scheibenbogen C, Sterry W and Keilholz U (1998) Long-term outcome of treatment with dacarbazine, cisplatin, interferon-α and intravenous high dose interleukin-2 in poor risk melanoma patients. *Melanoma Res* **8**: 557–563

Richards JM, Gale D, Metha N and Lestingi T (1999) Combination chemotherapy with interleukin-2 and interferon alfa for the treatment of metastatic melanoma. *J Clin Oncol* **17**: 651–657

Ronan SG, Eng AM, Briele HA, Shioura NN and Das Gupta TK (1987) Thin malignant melanomas with regression and metastases. *Arch Dermatol* **123**: 1326–1330

Rosenberg SA, Yang JC, Schwartzentruber DJ, Hwu P, Marincola FM, Topalian SL, Seipp CA, Einhorn JH, White DE and Steinberg SM (1999) Prospective randomized trial of the treatment of patients with metastatic melanoma using chemotherapy with cisplatin, dacarbazine, and tamoxifen alone or in combination with interleukin-2 and interferon-alpha 2b. *J Clin Oncol* **17**: 968–975

Si Z, Hersey P and Coates A (1996) Clinical responses and lymphoid infiltrates in metastatic melanoma following treatment with intralesional GM-CSF. *Melanoma Res* **6**: 247–255

Sondergaard K and Hou-Jensen K (1985) Partial regression in thin primary cutaneous malignant melanomas clinical stage I. *Virchows Arch A Pathol Anat Histopathol* **408**: 241–247

 *© 2001 Cancer Research Campaign*

Tefany FJ, Barnetson R St.C, Halliday GM, McCarthy SW and McCarthy WH (1991) Immunocytochemical analysis of the cellular infiltrate in primary regressing and non-regressing malignant melanoma. *J Invest Dermatol* **97**: 197–202

Thomas WD and Hersey P (1998) CD4 T cells kill melanoma cells by mechanisms that are independent of Fas (CD95). *Int J Cancer* **75**: 384–389

Zehntner S, Townsend W, Parkes J, Schmidt C, Down M, Bell J, Mulligan R, O'Rourke M, Ellem K and Thomas R (1999) Tumour metastasis biopsy as a surrogate marker of response to melanoma immunotherapy. *Pathology* **31**: 116–122

*© 2001 Cancer Research Campaign*
